# Antifungal Photodynamic Activity of Hexyl-Aminolevulinate Ethosomes Against *Candida albicans* Biofilm

**DOI:** 10.3389/fmicb.2020.02052

**Published:** 2020-09-11

**Authors:** Yingzhe Wang, Jinru Song, Feiyin Zhang, Kang Zeng, Xiaoliang Zhu

**Affiliations:** Department of Dermatology, Nanfang Hospital, Southern Medical University, Guangzhou, China

**Keywords:** *Candida albicans*, biofilm, photodynamic therapy, hexyl-aminolevulinate, ethosomes

## Abstract

Biofilm formation is responsible for the development of chronic and recurrent *Candida albicans* infections. The generation of biofilms is commonly accompanied by high resistance to conventional antifungal drugs, which can increase up to 1,000-fold. Fortunately, antimicrobial photodynamic therapy (aPDT) has shown excellent potential to treat biofilm infections. However, the current most commonly used photosensitizer (PS), aminolevulinic acid (ALA), is hydrophilic, unstable, and has low permeability, leading to unsatisfactory effects on biofilm eradication. To solve these problems, more stable lipophilic PSs and more effective permeability carriers could be considered as two effective solutions. Hexyl-aminolevulinate (HAL) has good bioavailability as a PS, and we proved in a previous study that ethosomes (ES), lipid-based nanocarriers, promote percutaneous drug penetration. In our previous study, a HAL-ES system presented superior photodynamic effects compared to those of ALA or HAL alone. Therefore, here, we aim to evaluate the biological effects of HAL-ES-mediated aPDT on *C. albicans* biofilm. An XTT sodium salt assay showed that aPDT using 0.5% HAL decreased *C. albicans* biofilm activity by 69.71 ± 0.43%. Moreover, aPDT with 0.5% HAL-ES further decreased biofilm activity by 92.95 ± 0.16% and inhibited growth of 25.71 ± 1.61% within 48 h, mostly *via* its effect on the hyphae growth, which correlated with a three-fold increase in *C. albicans* plasma membrane permeabilization. Notably, HAL-ES-mediated aPDT significantly reduced the sessile minimum inhibitory concentration 50 (SMIC50) of fluconazole to <2.0 μg/ml, and the 21-day survival rate of *C. albicans* biofilm-infected mice increased from 6.7 to 73.3%. It also significantly reduced the drug resistance and *in vivo* pathogenicity of *C. albicans* biofilm. These results demonstrate that HAL-ES-mediated aPDT could be an effective therapy for *C. albicans* biofilm infections; while also serving as a particularly promising effective treatment for cutaneous or mucocutaneous candidiasis and the prevention of progression to systemic candidiasis.

## Introduction

*Candida albicans* is a fungus naturally present in the skin and mucosa of healthy people, which can be pathogenic in case of systemic or local decline of immunity. Cutaneous candidiasis is a common disease affecting people of all ages, accounting for approximately 7.1% of patients with dermatosis (Penate et al., 2009). With the widespread use of antifungal drugs, drug resistance in *C. albicans* has become increasingly severe, especially considering azoles, such as fluconazole, while biofilm formation has been identified as the leading cause underlying *C. albicans* drug resistance (Holmes et al., 2012). Biofilms are composed of spores, pseudohyphae, and hyphae surrounded by a self-produced extracellular matrix (Wall et al., 2019). Microbes embedded in a biofilm can tolerate 100–1,000 times higher antibiotic concentrations than their corresponding free-living counterparts (Ceri et al., 1999). Since approximately 80% of human infections are related to biofilm formation, reduction or even elimination of biofilm is a major challenge in the treatment of fungal infections (Römling and Balsalbre, 2012).

Antimicrobial photodynamic therapy (aPDT) reportedly showed valuable therapeutic potential against biofilms (Baltazar et al., 2015; Hu et al., 2018). aPDT employs a photosensitizer (PS) accumulated in cells to generate, *via* irradiation with visible light, reactive oxygen species (ROS) that attack adjacent targets, including microbial pathogens and the biofilm matrix (Hu et al., 2018). In comparison with traditional antibiotic therapies, aPDT presents the advantages of reduced side effects, increased safety, and its efficacy is not affected by common microbial resistance mechanisms (Al-Mutairi et al., 2018). However, the current most commonly used PS, aminolevulinic acid (ALA), is hydrophilic and unstable, resulting in unsatisfactory penetration of biological barriers, such as plasma membranes; it also has low bioavailability, which significantly affects aPDT efficiency.

Hexyl-aminolevulinate (HAL) is a lipophilic PS. Compared to ALA, HAL has an additional hexyl group that confers extra membrane permeability and higher bioavailability (Togsverd-Bo et al., 2012). HAL offers the advantages of low therapeutic concentrations, few side effects, and better economic value (Neittaanmäki-Perttu et al., 2016). Besides, HAL-mediated PDT has contributed to the diagnosis and treatment of tumors and skin diseases, such as bladder cancer (Helander et al., 2016) and solar keratosis (Neittaanmäki-Perttu et al., 2017). However, its efficacy in biofilm elimination, which is crucial to the treatment of cutaneous candidiasis, has not yet been studied.

Ethosomes (ES) are an innovative vesicular delivery system with lipid bilayers. This system is composed of alcohol, phospholipids, and water, and has high encapsulation efficiency, small particle size, excellent stability, and low irritation potential. In our previous studies, we proved that ES more effectively promotes the penetration of lipophilic drugs into the deep layer of the skin through hair follicles and cuticles compared to liposomes and a hydroethanolic solution (Zhu et al., 2013; Yang et al., 2017). It can, therefore, be speculated that if HAL is effective in biofilm elimination, ES may boost the therapeutic effect, possibly even at lower HAL concentrations.

In the present study, we studied the photodynamic effect and the mechanism of HAL-ES in *C. albicans* biofilm, as well as its effects on fluconazole susceptibility and the *in vivo* pathogenicity of *C. albicans* biofilm.

## Materials and Methods

### Strains, Cultures, and Chemicals

*C. albicans* strain SC5314 was used in all experiments and was maintained on YPD agar (0.5% yeast extract, 1% bacto-peptone, 2% glucose, and 1.4% agar) plates at 37°C. HAL-ES at a 0.5% concentration was prepared by the injection-sonication-filtering method. Next, 0.12 g of soybean phospholipid (AVT, Shanghai, China) was mixed with 1.25 ml of ethanol to form an alcohol-based phase. In the aqueous phase, 20 mg of HAL (NMT Biotech, Suzhou, China) were dissolved in 2.75 ml of double-distilled water. The alcohol-based phase was slowly injected into the water phase (200 μl/min) under sealed conditions on a magnetic stirrer (700 rpm) for 5 min. The solution was then processed by an ultrasonic disruptor (JY92-IIN 650 W, Scientz, Ningbo, China) for 3 min at 97.5 W: the ultrasound was turned on for 5-s periods between 3-s intervals. The solution was then filter-sterilized and stored in the dark at 4°C for use within 30 min. The entire operation was performed in the dark. A blank solution of ES without HAL was prepared in the same way.

Solutions containing 0.5% HAL, 5% ALA, and 0.5% ALA were prepared in double-distilled water, filter-sterilized, and stored briefly in the dark at 4°C.

### Biofilm Preparation

A single colony of *C. albicans* was picked from the YPD agar medium using an inoculating loop, inoculated into a shake tube containing 5 ml of YPD liquid medium, and cultured overnight at 37°C with shaking at 200 rpm. The fungal suspension was centrifuged for 10 min (3,000 rpm, 4°C) and washed twice with sterile phosphate-buffered saline (PBS). The final pellet was resuspended in Gibco RPMI 1640 medium (Thermo Fisher Scientific, Waltham, MA, United States) and diluted to a cell density of 1.0 × 10^6^ cells/ml. Subsequently, 200 μl of the diluted fungal suspension was added to the selected wells of the 96-well plate, the plate was sealed with parafilm and incubated it at 37°C. After 24 h, samples were rinsed gently with PBS thrice to remove non-adherent planktonic cells. Finally, 200 μl of RPMI 1640 medium was added, and the incubation was continued for another 24 h.

### aPDT of Biofilms

To observe the effects of HAL-ES-mediated aPDT, *C. albicans* biofilms were treated with 0.5% HAL-ES, 0.5% HAL, 5% ALA, 0.5% ALA, blank ES, or PBS. Each well received 100 μl of PS, and the biofilms were incubated at 37°C for 30 min in the dark. The liquid in biofilms was then discarded, and wells were carefully rinsed thrice with sterile PBS to remove the residual drug. Next, 100 μl of sterile PBS was added to each well, and the biofilms were immediately irradiated with a light-emitting diode (LED) source (LED-IB PDT; Wuhan Yage Optic and Electronic Technique Co. Ltd., Wuhan, China) for 30 min (wavelength range: 633 ± 10 nm; power: 60 mW/cm^2^; irradiation distance: 10 cm). Finally, biofilms were incubated with RPMI 1640 medium in the dark for 0, 24, or 48 h before subsequent analysis.

### Biofilm Activity Assay

The evaluation of *C. albicans* biofilm activity was based on XTT (a tetrazolium salt) reduction by metabolically active fungal cells (Pierce et al., 2010). In each well, 100 μl of an XTT salt solution (Sigma-Aldrich, St. Louis, MO, United States; 0.5 mg/ml in PBS) and 1 μl of a menadione solution (10 mM in acetone) was applied, and the plate was incubated at 37°C in the dark for 2 h. Using a multichannel pipette, 80 μl of the resulting colored supernatant was removed from each well and transferred to the corresponding wells of a new 96-well plate. The plates were analyzed in a microplate reader (CLARIOstar Plus, BMG LABTECH, Ortenberg, Germany) at 490 nm.

### Visualization of Biofilm Structure

The structure of the *C. albicans* biofilm was observed by crystal violet staining. After methanol fixation, biofilms were stained with 0.1% crystal violet and left to dry overnight at room temperature. The observation was performed under an inverted microscope (IX71, Olympus, Tokyo, Japan).

### Fluorometric Estimation of Plasma Membrane Permeabilization

After aPDT and 0, 24, and 48 h of incubation, biofilms were carefully washed thrice with sterile physiological saline and were incubated in 0.2 ml of physiological saline containing 5 μM SYTOX Green (Molecular Probes Inc., Eugene, OR, United States) for 1 h at 37°C. The fluorescence intensity of each well was measured by a microplate reader. The excitation wavelength was 488 nm, and the emission range was 508–538 nm.

### Biofilm Testing for Fluconazole Susceptibility

Fluconazole (Sigma-Aldrich) was 2-fold serially diluted in RPMI 1640, from 1,024 to 2 μg/ml. Wells containing biofilm received 100 μl of each dilution, in addition to positive (no drug) and negative (empty wells) controls. After incubation at 37°C in the dark for 24–48 h, sessile minimum inhibitory concentration (SMIC) values in *C. albicans* biofilm were defined using the XTT assay as described above. SMIC50 and SMIC80 represent the antifungal concentrations at which we observed a 50 or 80% decrease in colorimetric readings in comparison to those in the positive control biofilms (Pierce et al., 2010).

### *In vivo* Pathogenicity of *C. albicans* Biofilm

Six‐ to eight-week-old BALB/c female mice (18–22 g) were used in this study. The mice were obtained from the Laboratory Animal Center, Nanfang Hospital, Southern Medical University, Guangzhou, People’s Republic of China. The animal use and care protocols were reviewed and approved by the Institutional Animal Care and Use Committee of Nanfang Hospital, Southern Medical University (NFYY-2019-71). All procedures of animal experiments adhered to the Principles of Laboratory Animal Care guidelines of the Laboratory Animal Center of Southern Medical University, following the principles of the Declaration of Helsinki. We evaluated *C. albicans* biofilm pathogenicity *in vivo* using a mouse model of systemic infection. Biofilms were subjected to aPDT, further incubated for 48 h, and finally detached by scraping the microplate surface with a sterile spatula. The biofilm suspension was then collected in a 50-ml centrifuge tube. The microplate was carefully washed twice with sterile PBS, which was then transferred to the same centrifuge tube. The suspension was centrifuged for 15 min (3,000 rpm, 4°C) and washed three times with sterile PBS. Finally, the biofilm suspensions of the control group were diluted to achieve an OD_600_ = 0.1, equivalent to 1 × 10^6^ cells/ml. Biofilm suspensions of the 0.5% HAL-ES, 0.5% HAL, 0.5% ALA, and blank ES groups were partly diluted at the same dilution ratio as that of the control group, partly diluted to OD_600_ = 0.1. Each group had 15 mice, which were injected with 0.1 ml of a thoroughly mixed biofilm suspension in their tail veins. The survival rates of mice were recorded every 24 h.

### Statistical Analysis

All results were obtained from three independent experiments, and each value was expressed as the mean ± standard deviation (SD). Statistical differences were evaluated by analysis of variance (ANOVA) and *post hoc* comparison with the Tukey-Kramer and Games-Howell tests. The survival data were analyzed by a log-rank (Mantel-Cox) test. *p* < 0.05 was considered significant.

## Results

### HAL-ES-Mediated aPDT Decreased *C. albicans* Biofilm Activity and Growth

**Figure 1:**
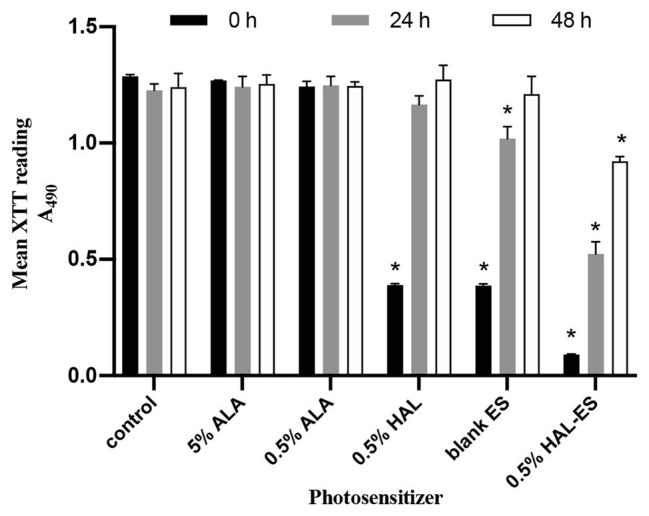
*C. albicans* biofilm activity after aPDT with different photosensitizers (PS). Data are presented as means ± SD (*n* = 3). ^*^p < 0.05 when compared to control.

After performing HAL-ES-mediated aPDT, we observed a sharp reduction in the activity and growth of *C. albicans* biofilm (Figure 1). Immediately after aPDT, when compared to the control group (100.00 ± 0.63%), 0.5% HAL-ES-mediated aPDT significantly reduced *C. albicans* biofilm activity (7.05 ± 0.16%, *p* < 0.001). Biofilm activity was also inhibited in the 0.5% HAL and blank ES groups (30.29 ± 0.43% and 30.10 ± 0.59%, respectively; *p* < 0.001). After 24 h, 0.5% HAL-ES-mediated aPDT significantly inhibited the growth of *C. albicans* biofilm (42.67 ± 4.24%) compared to the control group (100.00 ± 2.25%, *p* < 0.001). The blank ES group also had slightly decreased biofilm growth (83.15 ± 4.12%, *p* < 0.01). Biofilm activity in the 0.5% HAL group was restored to the same level as that in the control group (95.03 ± 3.06%, *p* > 0.05). After 48 h, when compared to the control group (100.00 ± 4.74%), 0.5% HAL-ES-mediated aPDT still inhibited the growth of *C. albicans* biofilm (74.29 ± 1.61%, *p* < 0.01). The biofilm activity in the blank ES group was restored to control levels (94.90 ± 10.11%, *p* > 0.05). Moreover, the biofilm activity of the 0.5% HAL group was not statistically different from the control group (102.58 ± 4.97%, *p* > 0.05). Similarly, the biofilm activity of the 5 and 0.5% ALA groups was not statistically different from the control group at 0 h (98.65 ± 0.09% and 96.63 ± 1.75%, respectively; *p* > 0.05), 24 h (101.25 ± 3.72% and 101.74 ± 3.23%, respectively; *p* > 0.05) and 48 h (101.07 ± 3.15% and 100.38 ± 1.41%, respectively; *p* > 0.05).

### HAL-ES-Mediated aPDT Reduced *C. albicans* Biofilm Formation

**Figure 2:**
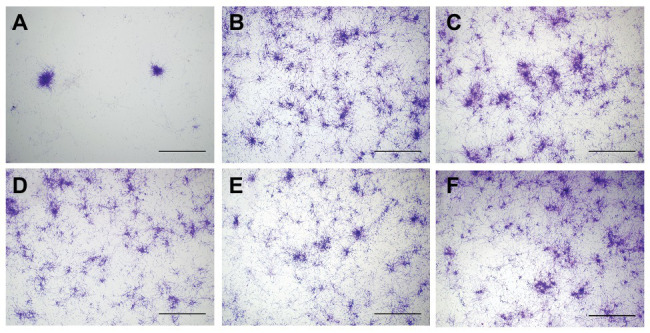
*C. albicans* biofilm morphology at 24 h after aPDT(×40). **(A)** 0.5% HAL ES, **(B)** 5% ALA, **(C)** 0.5% ALA, **(D)** 0.5% HAL, **(E)** blank ES, and **(F)** control. Bars: 100 μm.

At 24 h after aPDT, biofilm morphology was observed by an inverted microscope (Figure 2). The structure of biofilm formation was very different between the HAL-ES group and the other experimental groups. Biofilm formation in the HAL-ES group showed a few scattered larger microcolonies with rare hyphal connections (Figure 2A). The 5% ALA (Figure 2B), 0.5% ALA (Figure 2C), 0.5% HAL (Figure 2D), blank ES (Figure 2E), and control groups (Figure 2F) showed a large number of *C. albicans* microcolonies, and the hyphae between colonies were densely woven into an extensive network.

### HAL-ES-Mediated aPDT Increased Plasma Membrane Permeabilization in *C. albicans* Biofilms

**Figure 3:**
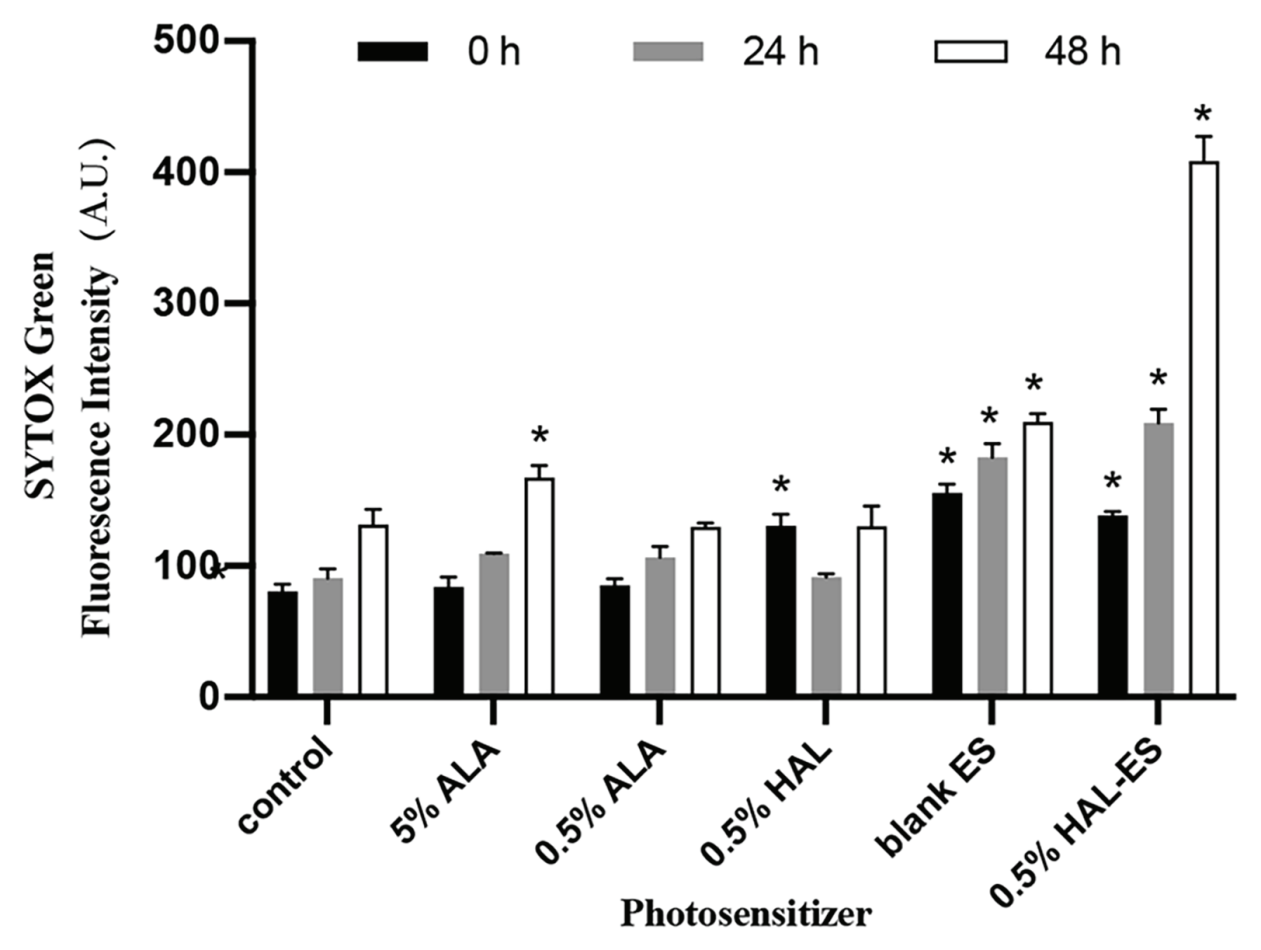
Plasma membrane permeability in biofilms after aPDT. Data were presented as means ± SD (*n* = 3). ^*^p < 0.05 as compared to control.

To further investigate the mechanism of HAL-ES-mediated aPDT on *C. albicans* biofilms, we used SYTOX Green to study plasma membrane permeability (Figure 3). SYTOX Green is a high-affinity fluorescent nucleic acid stain that penetrates cells with compromised plasma membranes, but not those with intact membranes (Giroldo et al., 2009).

Immediately after aPDT, the 0.5% HAL-ES, 0.5% HAL, and blank ES groups showed increased fluorescence intensity (138.33 ± 3.055, 130.67 ± 8.505, and 155.67 ± 6.506, respectively) compared to the control group (80.67 ± 5.508, all *p* < 0.001). At 24 h after aPDT, the fluorescence intensity in the 0.5% HAL-ES and blank ES groups increased (209.00 ± 10.583 and 182.67 ± 10.263, respectively) when compared to the control group (90.67 ± 7.234, *p* < 0.001), but the fluorescence intensity of the 0.5% HAL group was restored to that of the control group (91.33 ± 2.517, *p* > 0.05). After 48 h, the 0.5% HAL-ES group had the highest fluorescence intensity (408.67 ± 18.556, *p* < 0.001) compared to the control group (131.67 ± 11.372), and the blank ES group presented a slightly increased fluorescence intensity (209.67 ± 6.429, *p* < 0.001). At different incubation periods, the fluorescence intensities of the 5 and 0.5% ALA groups were almost the same as that of the control.

### HAL-ES-Mediated aPDT Increased the Susceptibility of *C. albicans* Biofilms to Fluconazole

As shown in Table 1, in the 0.5% HAL-ES group, the SMIC50 values were less than 2.0 μg/ml at 0, 24, and 48 h after aPDT, whereas the SMIC80 values were 4.0, 8.0, and >1,024 μg/ml, respectively. The SMIC50 and SMIC80 values of the 0.5% HAL and blank ES groups were equal to those of the 0.5% HAL-ES group at 0 h after aPDT, but with evident biofilm formation, the SMIC50 values were both greater than 1,024 μg/ml at 48 h after aPDT. In the 0.5% ALA group, the SMIC50 values were >1,024 μg/ml at 0, 24, and 48 h after aPDT.

**Table 1:** Fluconazole antifungal susceptibility of *Candida albicans* biofilm after aPDT.

Time after aPDT	SMIC (μg·ml^−1^)	Control	0.5%ALA	0.5%HAL	Blank ES	0.5% HAL-ES
0 h	SMIC 50	>1,024	>1,024	<2	<2	<2
	SMIC 80	>1,024	>1,024	4	4	4
24 h	SMIC 50	>1,024	>1,024	32	16	<2
	SMIC 80	>1,024	>1,024	128	128	8
48 h	SMIC 50	>1,024	>1,024	>1,024	>1,024	<2
	SMIC 80	>1,024	>1,024	>1,024	>1,024	>1,024

SMIC50 and SMIC80 were read as the lowest concentrations that produced a 50 or 80% reduction in growth compared with that of the drug-free control. aPDT, antimicrobial photodynamic therapy; ALA, aminolevulinic acid; HAL, hexyl-aminolevulinate; ES, ethosomes.

### HAL-ES-Mediated aPDT Weakened the *in vivo* Pathogenicity of *C. albicans* Biofilm

**Figure 4:**
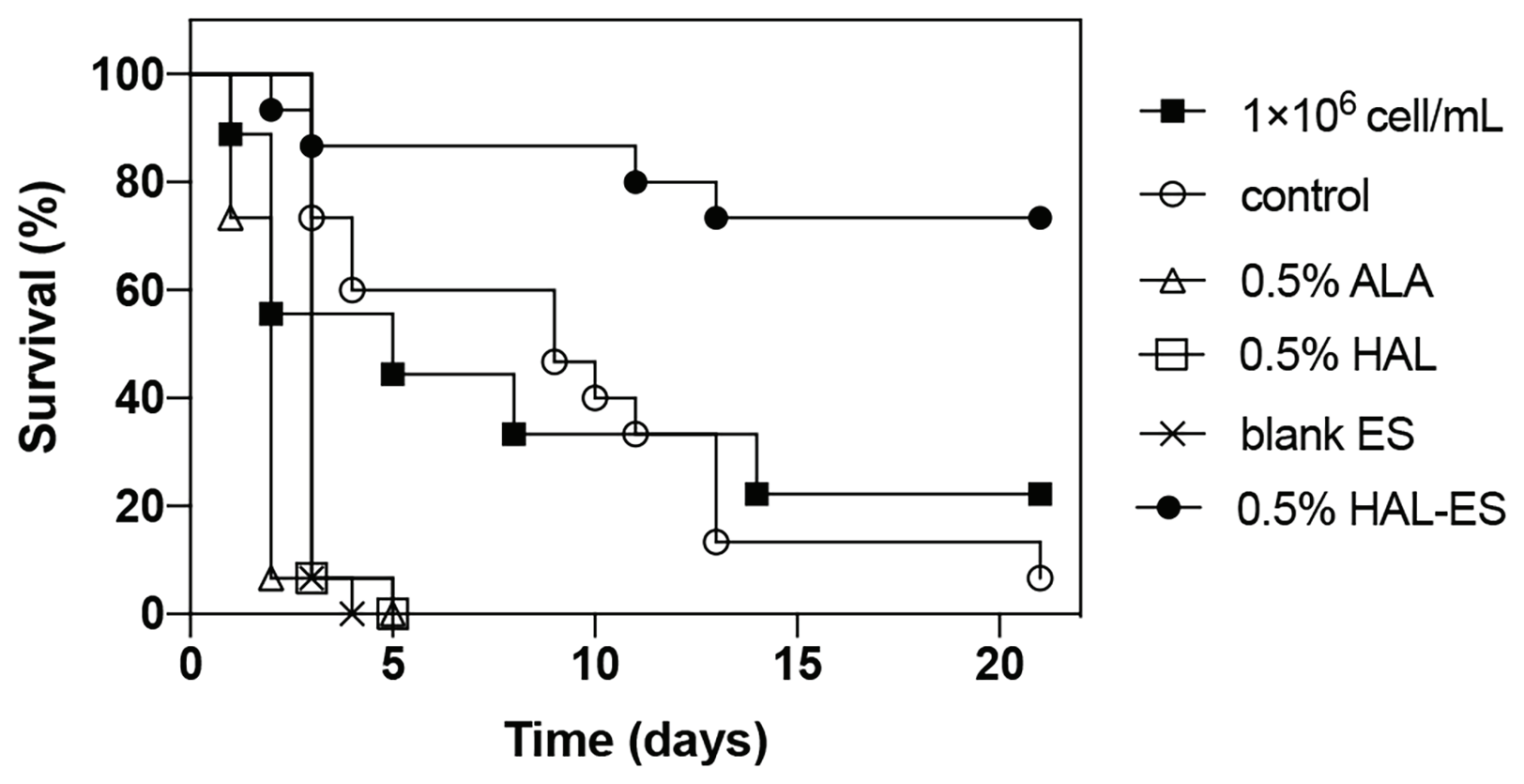
Survival curves for mice systemically infected with *C. albicans* biofilm at the same dilution. Results are representative of 1 × 10^6^ cell/ml *C. albicans* planktonic cultures (*n* = 9) and biofilm suspensions (*n* = 15).

**Figure 5:**
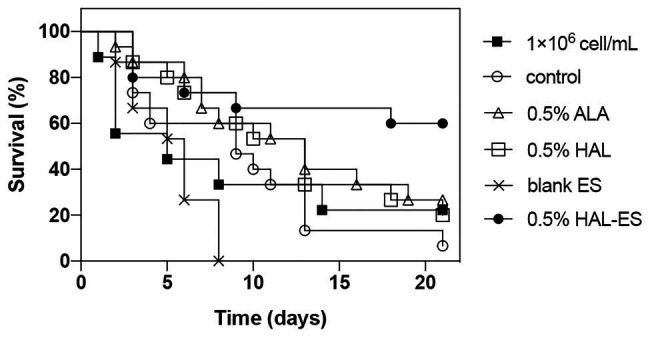
Survival curves for mice systemically infected with *C. albicans* biofilm with the same OD_600_ value. Results are representative of 1 × 10^6^ cell/ml *C. albicans* planktonic cultures (*n* = 9) and biofilm suspensions (*n* = 15).

Compared with the control, the survival rate (Figures 4, 5) of the 0.5% HAL-ES group was significantly improved (21-day survival rate at the same biofilm dilution factor and the same biofilm OD_600_ value is 73.3 and 60%, respectively; *p* = 0.0004 and *p* = 0.0055). The survival rate of the control group when using *C. albicans* biofilm suspension was not significantly different from when using 1 × 10^6^ cells/ml planktonic cultures (21-day survival of 6.7 and 22.2%, respectively; *p* > 0.05). Furthermore, when the biofilm was at the same dilution factor, all mice in the 0.5% ALA, 0.5% HAL, and blank ES groups died within 5 days. Meanwhile, when the biofilm was at the same OD_600_ value, the survival rates of the 0.5% ALA and 0.5% HAL groups were not significantly different from the control group (21-day survival of 26.7 and 20%, respectively; *p* > 0.05), and all mice in the blank ES groups died within 8 days.

## Discussion

aPDT has been proven to inactivate biofilm effectively and is considered a promising alternative to antifungal drugs. This treatment first employs a PS topically to a confined area and then irradiates it with a specific light wavelength capable of exciting the PS to induce production of cytotoxic ROS in the presence of ambient molecular oxygen, which subsequently causes cell death by disruption of macromolecules (e.g., DNA, RNA, and proteins), and membranes. However, aPDT requires more effective PS and carriers to improve biofilm eradication efficiency (Hu et al., 2018). This study has shown that, compared to 5 and 0.5% ALA-mediated aPDT, 0.5% HAL-mediated aPDT inhibited *C. albicans* biofilm activity due to the higher bioavailability of HAL (Togsverd-Bo et al., 2012). Moreover, 0.5% HAL-ES-mediated aPDT further significantly inhibited the activity and growth of *C. albicans* biofilm within 48 h, mainly due to the effective reduction of biofilm hyphae. This can be because ES, as a nanocarrier, contains a high concentration of ethanol and is of reduced size, allowing the drug to effectively penetrate the structure of biofilm and cell membranes (Zhang et al., 2014; Niu et al., 2019). Hyphae play a pivotal role in the spatial structure and compressive strength of biofilms (Paramonova et al., 2009). Targeting hyphae formation can thus significantly weaken *C. albicans* biofilm drug resistance. Furthermore, aPDT can directly damage the biofilm through ROS, thereby physically disrupting the colony aggregation process (Fila et al., 2016). Finally, aPDT can trigger changes in genes related to the morphological transformation of the *C. albicans* yeast phase to its hyphal form (Paramonova et al., 2009), which are essential for biofilm formation (Mayer et al., 2013). In contrast, 5 and 0.5% ALA-mediated aPDT had no significant effects on *C. albicans* biofilm under our experimental conditions, which was consistent with previous studies (Monfrecola et al., 2004; Shi et al., 2016).

The mechanism underlying HAL-ES-mediated aPDT antimicrobial effect might be mediated by the physical destruction of microbial structures, such as plasma membranes (Hamblin, 2016). Our results showed that damage to the plasma membrane in viable *C. albicans* treated with HAL-ES-mediated aPDT was more significant than that observed in the 0.5% HAL and blank ES groups. With the growth of *C. albicans* biofilm, the SYTOX Green fluorescence intensity in the 0.5% HAL-ES group tripled at 48 h after aPDT, suggesting that plasma membrane damage gradually accumulated and eventually led to inhibition of *C. albicans* growth and biofilm formation (Giroldo et al., 2009). On the other hand, 5 and 0.5% ALA had almost no effects on the plasma membrane, which could be that HAL is lipophilic, while ALA is hydrophilic, allowing easier penetration of HAL through the lipid bilayer, which ensures more prominent photodynamic effects that destabilize the plasma membrane. Meanwhile, membrane permeability also increased in the blank ES group, which could be due to the ethanol and small particle sizes of ES. It shall be highlighted that the damage caused by HAL-ES-mediated aPDT to the plasma membrane exceeded the additive effects of HAL and ES treatments, which could be because, under the same conditions, ES permits the deeper penetration of HAL into the biofilm.

Increased plasma membrane permeability facilitates entry and accumulation of drugs into the cell and ultimately plays an antifungal role, which in this study was reflected by a reduction in *C. albicans* biofilm drug resistance (Holmes et al., 2012). Our results showed that aPDT mediated by 0.5% HAL-ES, 0.5% HAL, and blank ES enhanced fluconazole susceptibility in *C. albicans* biofilm, which is related to the reduced biofilm activity after aPDT, illustrating the effectiveness of combining aPDT and fluconazole, after all, the highest dose of fluconazole elicited negligible effects against the activity of the *C. albicans* biofilm. Moreover, 0.5% HAL-ES-mediated aPDT more effectively sensitizes biofilms to fluconazole than 0.5% HAL or blank ES even if the fluconazole susceptibility gradually weakened as the biofilm grew, suggesting that in clinical treatment, earlier administration of aPDT combined with fluconazole, will require lower doses of fluconazole to achieve satisfactory antifungal effects. The reduced fluconazole susceptibility may also be explained by other mechanisms, such as the promotion of phenotypic changes involving the drug resistance-related genes *MDR1*, *QDR1*, and *ERG* (Wall et al., 2019).

The *in vivo* pathogenicity of biofilms in the HAL-ES group was the lowest of all groups. This is due to the inhibition of biofilm growth and the increased permeability of the plasma membrane. Interestingly, the *in vivo* pathogenicity of biofilms in the 0.5% ALA, 0.5% HAL, and blank ES groups was increased when compared to that in the control group. This result could be due to increased biofilm formation and mutagenesis caused by the stress response (Jolivet-Gougeon and Bonnaure-Mallet, 2014). Previous studies have shown that sub-lethal photodynamic therapy and a low ethanol concentration can increase the biofilm formation ability (Kashef et al., 2013; Pourhajibagher et al., 2016; Tango et al., 2018), and an antimicrobial treatment can induce 105 times more biofilm variability than that observed in free-living cultures (Driffield et al., 2008). Notably, while the fluconazole susceptibility of *C. albicans* biofilm decreased and pathogenicity increased in the 0.5% HAL and ES groups, biofilm pathogenicity was significantly weakened in the 0.5% HAL-ES group, suggesting that aPDT requires an efficient PS to reduce *C. albicans* biofilm pathogenicity.

In conclusion, this study proved that HAL-ES-mediated aPDT had more potent antifungal photodynamic activity than did HAL-mediated, and ALA-mediated aPDT. Its mechanism of action could be related to the inhibition of *C. albicans* growth and biofilm formation, as well as the reduction of drug resistance and *in vivo* pathogenicity. HAL-ES-mediated aPDT is, therefore, a promising novel treatment against *C. albicans* biofilms. Moreover, ES is a nanoparticle with excellent percutaneous penetration efficiency. In our previous studies, it was confirmed that its percutaneous flux is much higher than that of liposomes and hydroethanolic solution (Zhu et al., 2013; Yang et al., 2017). Therefore, HAL-ES-mediated aPDT can offer an alternative treatment for patients with cutaneous or mucosal *C. albicans* biofilm infections. It is particularly suitable for patients for whom topical antifungal agents have been proven ineffective, or who have contraindications to oral antifungal drugs. Lastly, HAL-ES-mediated aPDT can also function synergistically with fluconazole treatment to prevent local candidiasis from developing into a systemic infection.

## Data Availability Statement

All datasets presented in this study are included in the article/supplementary material.

## Ethics Statement

The animal study was reviewed and approved by The Institutional Animal Care and Use Committee of Nanfang Hospital, Southern Medical University (NFYY-2019-71).

## Author Contributions

XZ and YW designed the experiments. YW performed most of the experiments. JS participated in the animal experiments. FZ participated in data analysis. XZ, KZ, and YW wrote the manuscript. All authors contributed to the article and approved the submitted version.

### Conflict of Interest

The authors declare that the research was conducted in the absence of any commercial or financial relationships that could be constructed as a potential conflict of interest.
